# Diffractive lenses for neutron techniques

**DOI:** 10.1038/s41598-025-92329-6

**Published:** 2025-03-11

**Authors:** Mano raj Dhanalakshmi Veeraraj, Di Qu, Shuai Zhao, Peng Qi, Konstantins Jefimovs, Matteo Busi, Joachim Kohlbrecher, Christian David, Markus Strobl, Joan Vila-Comamala

**Affiliations:** 1 PSI Center for Photon Science, Paul Scherrer Institut, 5232 Villigen PSI, Switzerland; 2 PSI Center for Neutron and Muon Sciences, Paul Scherrer Institut, 5232 Villigen PSI, Switzerland; 3 Niels Bohr Institute, University of Copenhagen, 2100 København, Denmark; 4 National Synchrotron Radiation Laboratory, University of Science and Technology of China, Hefei, 230029 China

**Keywords:** Neutron microscopy, Fresnel zone plate, Full-field neutron imaging, SANS, Techniques and instrumentation, Optics and photonics

## Abstract

Many neutron techniques can greatly benefit from enhanced neutron lenses for focusing and imaging. In this work, we revisit the potential of diffractive optics for neutron beams, building on advanced high-resolution nano-lithography techniques developed for the fabrication of X-ray diffractive optics used at synchrotron facilities. We demonstrate state-of-the-art fabrication of nickel and silicon Fresnel zone plates and we report proof-of-concept experiments for full-field neutron microscopy and small angle neutron scattering. The advancement of neutron diffractive optics will open new opportunities for neutron techniques, improving both the efficiency and resolution of existing instruments.

## Introduction

Neutron and X-ray techniques are complementary for non-destructive material analysis^1,2^. While X-rays interact with the atomic electron cloud providing elemental and chemical sensitivity, the charge-less neutrons interact with the nucleus thus providing high penetrability and isotope sensitivity. Neutrons and X-rays are also complementary in the sense that elements of high atomic number will attenuate X-rays to a great extent, whereas neutrons are strongly absorbed by some light elements of low atomic number but penetrate well some materials of heavy elements. Thus, the combination of X-rays and neutrons enables a more complete analysis of bulk material of different elements^3^.

Numerous types of optical devices have been demonstrated for neutron imaging and other techniques that rely on neutron focusing. These optical elements are based on reflection^4,5,7^, refraction^8–12^ diffraction^13,14^ or even magnetism^15–17^. The development of diffractive optics^18–21^ has found many applications for X-ray microscopy and other methods requiring X-ray focusing. Nowadays, high-quality X-ray diffractive optics can be produced by high-resolution lithography combined with several nanofabrication methods such as metal electroplating and reactive ion etching. These diffractive elements can be made out of materials such as silicon, diamond, gold or nickel depending on the particular requirements^22–25^. For neutron techniques, even though diffractive optics were proposed and demonstrated^13,14^, their potential has not been fully exploited. For example, diffractive lenses could be used to realize neutron full-field microscopy, in which the entire volume of the object is illuminated by the neutron beam, and an objective lens is placed behind the object to produce a magnified image on the detector. To date, such schemes are not commonly used for neutrons due to the lack of suitable neutron lenses. In addition to imaging, diffractive optics can be used in scattering instruments for beam delivery and focusing. The performance of an instrument with such optics critically relies on the efficiency of the optics, as well as the level of diffuse background generated by them. Despite attempts to develop neutron lens-based instruments, pinhole collimation setups are still commonly used in neutron imaging and small angle neutron scattering (SANS) beamlines.

Here, we demonstrate that the advanced nanofabrication methods for producing X-ray diffractive optics can be readily extended to enable diffractive optics for neutron-based applications. We report on the design, fabrication, and characterization of diffractive lenses for cold neutrons. We also show experimental results using these components for full-field neutron microscopy and small-angle neutron scattering.

## Fresnel zone plate theory

In the following section, we describe the essential design rules for the Fresnel zone plate (FZP), based on the interaction of radiation with material of the diffractive element. For both neutron and X-rays, their interaction with matter can be described by the material’s complex refractive index, $n = 1 - \delta + i\beta$, where $\delta$ and $\beta$ respectively account for the phase shift and absorption of the radiation beam passing through the object. In the case of neutrons, *n* can be determined from

1 $$\begin{aligned} n = 1 - \frac{\lambda ^2 \rho b_c }{2 \pi } + \frac{i \lambda \rho \sigma _r}{4 \pi } \end{aligned}$$

where $\rho$ is the number of nuclei per unit volume, $b_c$ is the bound coherent scattering length, and $\sigma _r$ is the sum of absorption and incoherent scattering cross section^26^. Then, the phase shift $\Delta \Phi$ introduced by a material thickness $\Delta T$ at neutron wavelength $\lambda$ is given by

2 $$\begin{aligned} \Delta \Phi =\left( \frac{2\pi \delta }{\lambda } \right) \Delta T \end{aligned}$$

When designing and fabricating diffractive elements, it is essential to calculate the material thickness that corresponds to a $\pi$ phase shift, as this phase shift maximizes the efficiency for binary diffraction optical elements. The required thickness $\Delta T _\pi$, which produces a $\pi$ phase shift is given by

3 $$\begin{aligned} \Delta T_\pi = \frac{\lambda }{2\delta } \end{aligned}$$

Another key parameter in selecting the appropriate material is the linear attenuation coefficient, $\mu$, as excessive absorption will greatly reduce the efficiency of the diffractive optical element. The material’s linear attenuation coefficient $\mu$ can be determined as

4 $$\begin{aligned} \mu = \frac{4\pi \beta }{\lambda } \end{aligned}$$

**Table 1:** Neutron optical parameters at 4.5 Å  wavelength for nickel, silicon, diamond, gold and quartz. With the exception of quartz other materials are commonly used for the nanofabrication of X-ray diffractive optics.

	$$\delta \,\, (\times 10^{-6})$$	$$\mu$$ (cm$$^{-1})$$	$$\Delta T_{\pi }$$ ($$\upmu$$m)
Nickel	30.3	2.716	7.43
Silicon	6.68	0.130	33.7
Diamond	37.8	0.980	5.95
Gold	14.5	15.027	15.5
Quartz	13.5	0.294	16.7

Typically, $\delta$ is of the order $\approx 10^{-6}$ as shown in Table 1, and $n<1$ for most elements. The neutron absorption of the commonly nanopatterned materials for X-ray diffractive optics^22–25^, with the exception of gold, is very low. The ideal material for diffractive optics should have a high value of $\delta /\mu$ to obtain the highest diffraction efficiency. Therefore nickel, silicon, and diamond are good candidates for diffractive neutron optics. Apart from the materials that are commonly nanopatterned for X-ray diffractive optics, quartz has a high value of $\delta /\mu$. But patterning high aspect ratio nanostructures is comparatively more challenging.

**Fig. 1:**
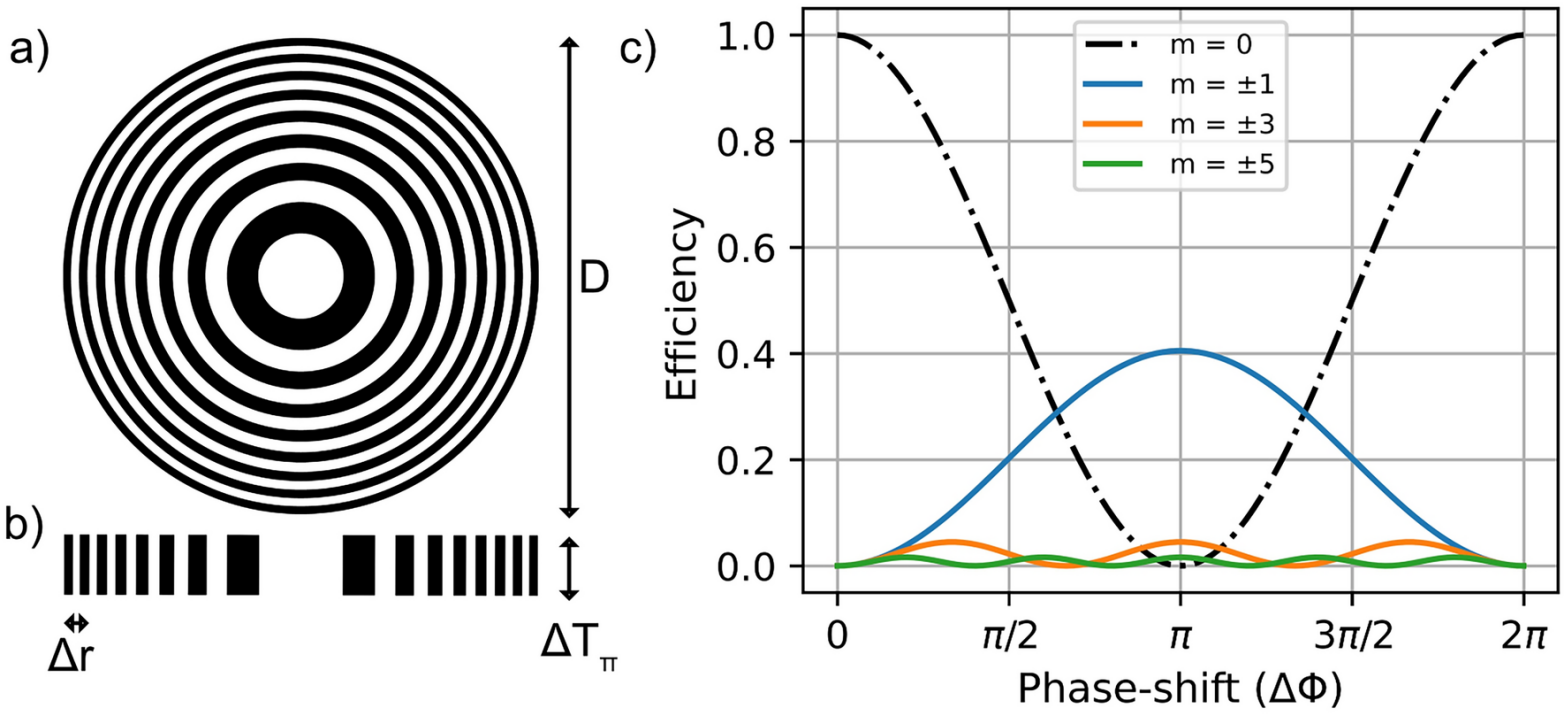
(**a**) Scheme of a Fresnel zone plate (FZP) of diameter *D*. (**b**) Cross section of the FZP showing the outermost zone width $\Delta r$ and the thickness $\Delta T_{\pi }$. (**c**) Efficiency of several diffraction orders ($\pm 1,\pm 3$ and $\pm 5$) of binary FZP as a function of the phase shift. The black dashed line corresponds to the zeroth order, that is, the direct beam transmitted through the FZP.

A FZP^27,28^, as schematically shown in Fig. 1a and its cross section in Fig. 1b, is a diffractive optical element that can be used for focusing and imaging. Considering the ideal case of no absorption and for obtaining the highest diffraction efficiency at 1*st* order, it consists of an alternating array of $\pi$-phase shifting and non-phase shifting rings, also referred as zones, with radially decreasing line widths. For radiation of wavelength $\lambda$, the focal length of a FZP is given by

5 $$\begin{aligned} f_{FZP} = \frac{D \Delta r}{\lambda } \end{aligned}$$

where *D* and $\Delta r$ are respectively the diameter and the width of the outermost zone of the FZP. It can also be shown that the Rayleigh criterion for diffraction-limited spatial resolution $\delta _r$ for an FZP can be expressed as

6 $$\begin{aligned} \delta _r = 1.22 \Delta r \end{aligned}$$

Because the focal length is inversely proportional to the wavelength, FZPs suffer from chromatic aberration when broadband radiation is used. Given a finite spectral bandwidth, $\Delta \lambda$, the condition^27,28^ for achieving the diffraction-limited spatial resolution $\delta _r$ is

7 $$\begin{aligned} \frac{\Delta \lambda }{\lambda } \le \frac{1}{N} \end{aligned}$$

where *N* is the total number of zones of the FZP. As typical FZPs have hundreds or even thousands of zones, the required narrow-band radiation usually cannot be provided at neutron sources. Thus, FZPs for neutron methods will not achieve diffraction-limited spatial resolution due to their chromatic aberration.

The low flux of neutron sources requires highly efficient FZPs. The optimal thickness of the nanostructures in an FZP depends on the $\Delta T_{\pi }$ of the material of choice at the operational wavelength $\lambda$, typically microns or tens of microns. On the other hand, due to the typical available neutron instrument length of $\approx$ 10 m, short focal lengths of the FZP are required, $f_{FZP}<<L/4$ of about 1 m, which in turn requires outermost zone width, $\Delta r$ of a few hundreds of nanometers. As a result, high aspect ratio nanostructures are required, where the aspect ratio is defined as

8 $$\begin{aligned} AR = \frac{\Delta T}{\Delta r} \end{aligned}$$

An ideal $\pi$-phase shifting FZP with equal width of the phase shifting and non-phase shifting zones, that is with a $50 \%$ duty cycle, has no direct beam (zeroth order beam) on the detector. A $\pi$-phase FZP without absorption delivers $40.5 \%$ of the incoming radiation to the focus of $+1st$ diffraction order and another $40.5 \%$ to the focus of the $-1st$ diffraction order and the rest to the higher diffraction orders as shown in Fig. 1c.

## Fresnel zone plates for full-field neutron microscopy

Commonly in a neutron imaging beamline, the object of interest is close to the detector and the pinhole upstream optimized for minimal blur involving a compromise between the resolution and the exposure time. In such an instrument, the resolution is intrinsically limited by the resolution of the detector. To overcome this fundamental limitation, a magnifying objective lens is needed.

### Design and simulation of Fresnel zone plates for full-field microscopy

**Fig. 2:**
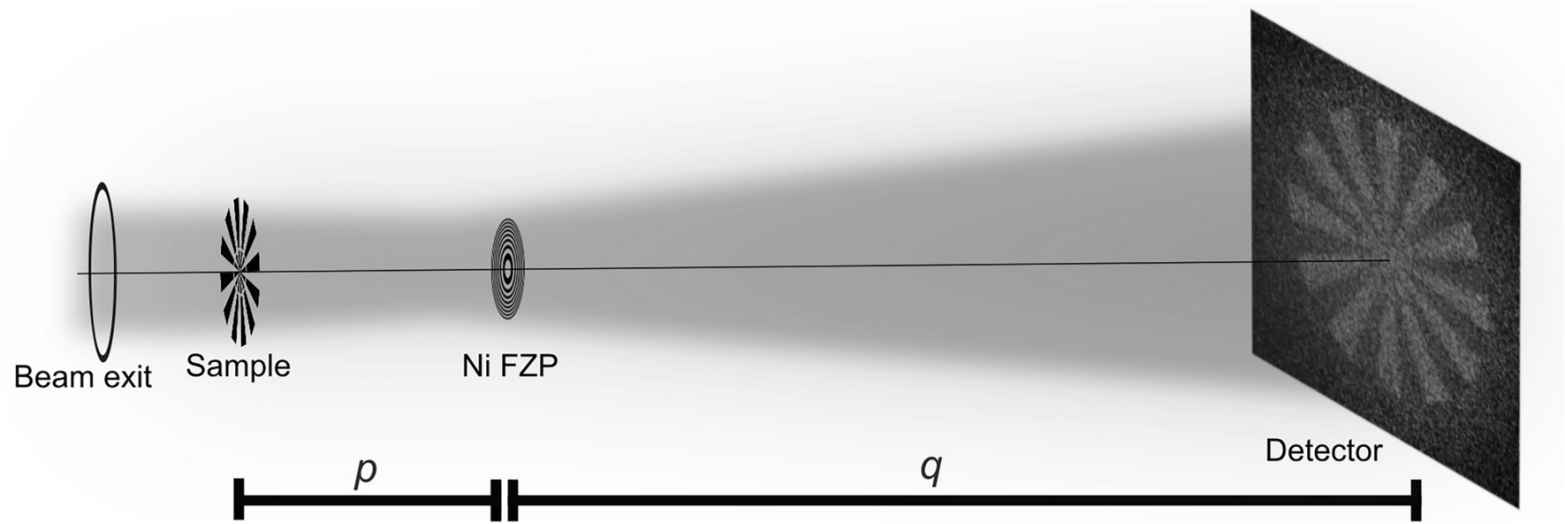
Schematic of a full-field neutron microscope setup with an FZP as the objective lens, producing a magnified image of the sample on the detector.

In a full-field microscope geometry with an objective lens, the object is positioned upstream and the lens is placed between the object and the detector to produce a sharp magnified image on the detector. Most neutron imaging beamlines around the world today are designed for the pinhole geometry with $\approx$ 8 m hutch length. These limitations impose challenges in realizing a lens, as short focal lengths need to be realized to achieve reasonable magnification. Similar to high resolution X-ray microscopy, we designed the FZP as the objective lens as shown in Fig. 2.

**Fig. 3:**
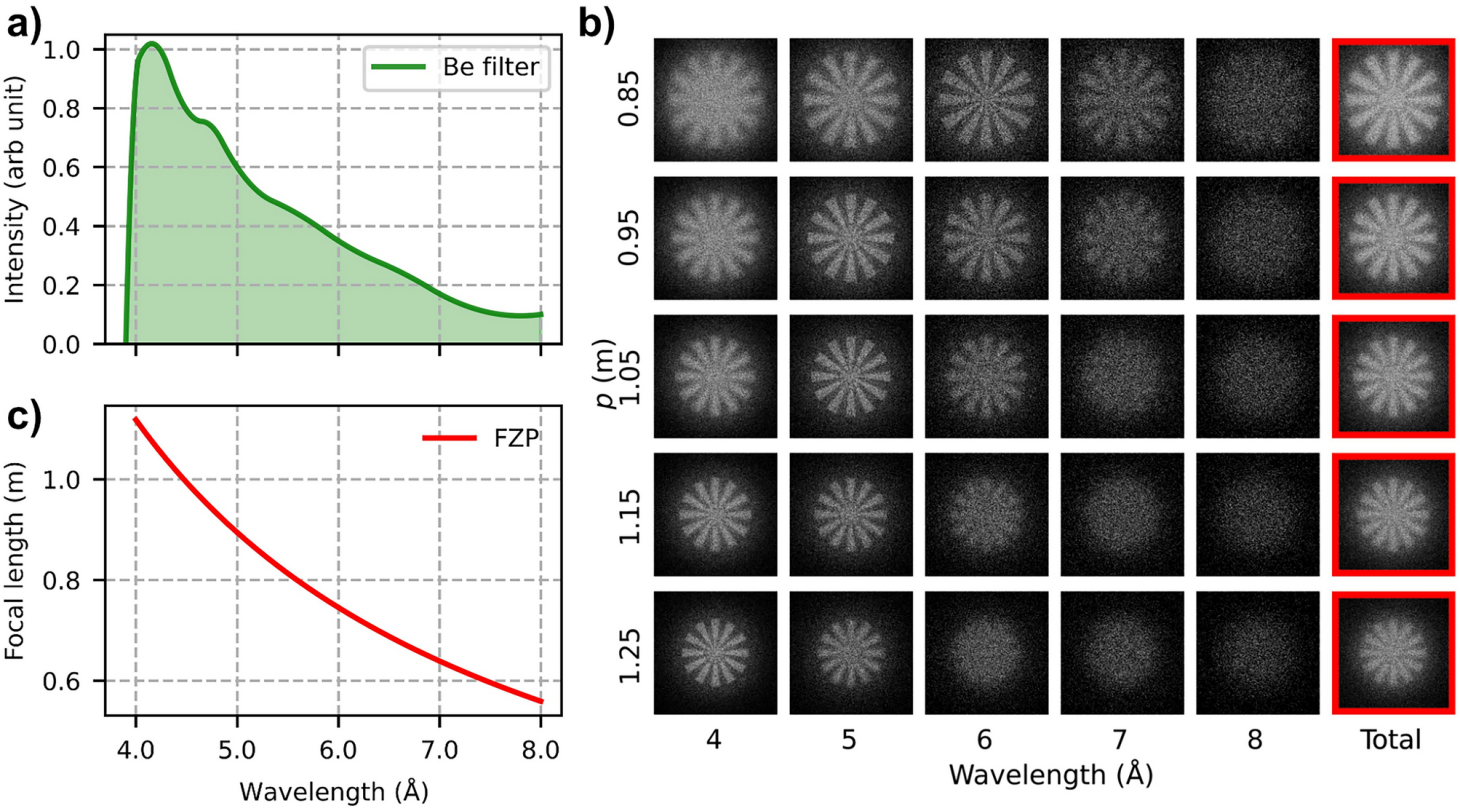
(**a**) Cold neutron spectrum of the BOA beamline with a beryllium filter. (**b**) Simulated focus scan matrix at different wavelengths using McStas simulation package, the individual wavelength bins are weighted according to the cold spectrum of BOA, SINQ with the beryllium filter. The effective image as a sum of all wavelengths (4–8 Å) is highlighted in red and labeled as Total. (**c**) Focal length as a function of the wavelength for an FZP with a diameter of 1.2 mm and an outermost zone width of 372 nm.

Depending on the design wavelength and the focal length needed to produce the required magnification within the limits of the beamline, the diameter of the FZP, $\Delta T$, and $\Delta r$ are determined. Our full-field neutron microscopy experiments with an FZP were planned at the BOA beamline of the SINQ spallation source at the Paul Scherrer Institut (Switzerland). The neutron flux at the BOA beamline is distributed around 3 Å^29^. A beryllium filter cooled to $\approx$ 60 K can be used to filter out shorter wavelength neutrons ($\lambda<$ 4.05 Å)^30^, resulting in an effective spectrum with a weighted average at $\approx$ 4.5 Å  as shown in Fig. 3a. At this wavelength, the highest efficiency can be obtained for about a $\pi$-shift thickness of $7.4\,{\upmu }\hbox {m}$ of nickel. In addition, the lens was optimized and designed for the conditions of the BOA beamline with the beryllium filtered beam, with a design focal length of $\approx$ 0.99 m to enable a magnification of about $7\times$ within the total available 8 m instrument length. The diameter of the FZP was chosen as 1.2 mm and as a result, an outermost zone width of about 372 nm was required. More details about the parameters of the nickel FZP used are given in Supplementary Table 1.

McStas simulations^31–33^ were performed to estimate the broad-band imaging performance of the FZP, using the mask component with a binary image of the resolution target, and the FZP_simple component. The bandwidth in each of the wavelength bins was chosen to be ± 0.5 Å  and was weighted according to the flux distribution of the BOA beamline. Despite the chromatic aberration of the FZP as shown in Fig. 3c, flux intensity decreasing towards longer wavelengths allows the broadband image, to still have enough contrast to be able to resolve high-frequency features as shown in Fig. 3b. It should also be noted that the detector component used in the simulations is perfect and does not introduce additional blur as in the case of a thick scintillator.

### Fabrication of FZPs for full-field neutron microscopy

The FZP material and the fabrication technique are chosen depending on the complexity of different lithography techniques in achieving the above design parameters. Gold and nickel nanostructures can be made by electroplating in the resist patterned by electron-beam lithography^18,34^. Diamond and silicon structures can be made by deep reactive ion etching (DRIE) techniques using the electron-beam patterned resist or metal layer as a mask^35^. The choice of material not only depends on $\delta /\mu$ but also on the technological limitations of these patterning processes. Sub 200 nm structures with $\Delta T < 2\,{\upmu }\hbox {m}$ are typically made with electroplating techniques. Coarser structures with larger dimensions can be made with very high $\Delta T >2\,{\upmu }\hbox {m}$ using silicon DRIE.

**Fig. 4:**
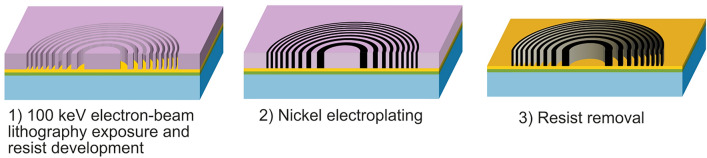
Fabrication steps of a nickel FZP by electron-beam lithography and electroplating.

**Fig. 5:**
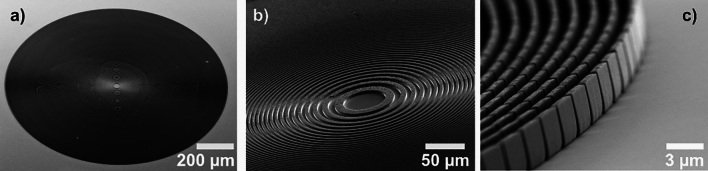
Scanning electron microscope (SEM) images of a high aspect ratio nickel FZP. (**a**) Overview image, (**b**) Zoom of the central zones, (**c**) Zoom of the outermost zones.

The FZPs were fabricated using electron-beam lithography following the process flow depicted in Fig. 4. Fused silica substrates with a size of 10 mm $\times$ 10 mm were coated with 5 nm thick Cr as an adhesion layer and 25 nm thick Au as a seed layer. The substrate was spin-coated with 11% PMMA 950K solution in anisole at 1500 rpm. After the spin-coating, the substrate is then baked at 175$^{\circ }$C for 5 minutes resulting in a thickness of $3\,{\upmu }\hbox {m}$. The pattern was exposed with a 100 keV electron beam using a Vistec EBPG 5000+ system. The exposed resist was developed at room temperature in a 7:3 IPA:H$_2$O solution. The resist mold was then filled with nickel by electroplating techniques. The SEM images of the 1.2 mm diameter nickel FZP are shown in Fig. 5.

At 4.5 Å,   the maximum diffraction efficiency of nickel corresponds to a $\Delta T_\pi =7.4\,{\upmu }\hbox {m}$. Electron scattering effects make the nanopatterning of a thick resist $( > 3 \ \mu \text {m} )$ challenging. To not compromise the efficiency, we stacked two FZP with $\Delta T_{exp}=3\,{\upmu }\hbox {m}$ nickel nanostructures with high precision using a laboratory X-ray source^36^. The proximity alignment conditions for near field stacking of FZP are met^37,38^, when the longitudinal distance (*d*) between the stacked FZPs is less than $0.76\Delta r^2/\lambda = 234\,{\upmu }\hbox {m}$ while maintaining a lateral misalignment between the FZPs of less than $\Delta r/3$ = 124 nm. Under such conditions, the stacked FZPs behave as a single optical element with $6\,{\upmu }\hbox {m}$ high structures. The resulting FZP stack with $6\,{\upmu }\hbox {m}$ thickness was limited to a theoretical diffraction efficiency of $\approx 36\%$ at the design wavelength as shown in Supplementary Fig. 1a.

### Full-field neutron microscopy experiments

**Fig. 6:**
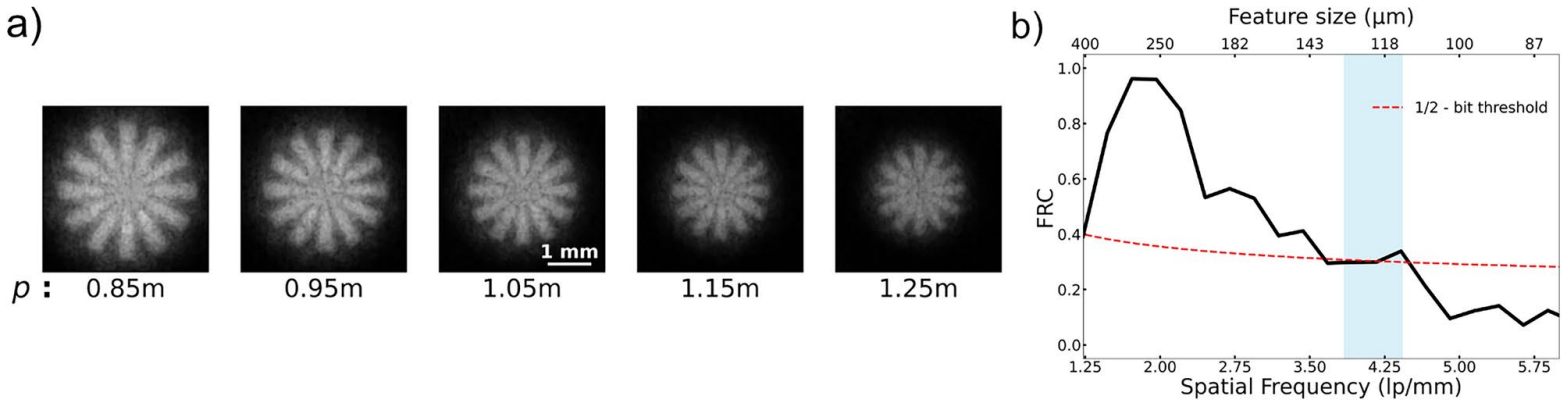
(**a**) Focal scan of a 3 mm diameter Gd Siemens star with FZP as the objective lens, (**b**) Fourier ring correlation calculated for the *p* =1.05 m image showing the resolution the of the image.

Imaging experiments using the nickel FZPs described above were performed at the BOA beamline^29^. The beryllium filtered neutron beam was collimated with a 40 mm pinhole at the beam exit. A highly absorbing resolution test pattern was positioned at 0.6 m downstream of the pinhole. It was a 3 mm diameter Gd Siemens star consisting of the largest structures ranging from $400\,{\upmu }\hbox {m}$ to the smallest structures being $8\,{\upmu }\hbox {m}$ as shown in Supplementary Fig. 2. The FZP with Cd aperture was positioned at $\approx$ 1 m downstream of the sample. An Andor IKON M CCD camera coupled with a $200\,{\upmu }\hbox {m}$ ZnS$:^{6}$LiF scintillator from RC Tritec, was positioned 8 m from the beam exit as shown in Fig. 2. A focal scan was performed by moving the lens from *p* = 0.85 m to 1.25 m in steps of 0.1 m as shown in Fig. 6a. 30 frames of 1 minute exposure time were acquired at every lens position, along with equivalent flat field images with no object in the beam and dark images with closed neutron shutter.

In data processing, first, a flat-field correction was performed to eliminate detector artifacts. Subsequently, multiple frames were combined using a weighted averaging method^39^, which has been shown to enhance SNR more effectively than pixel-wise median averaging and also remove outliers caused by gamma events. Finally, noise removal was accomplished using the Block-Matching and 3D Filtering (BM3D) algorithm, see Supplementary Fig. 2^40,41^. The resolution of the image was assessed using Fourier ring correlation (FRC)^42,43^ as shown in Fig. 6b. The 30 frames of images acquired were divided into two sets of 15 frames and used to calculate the FRC. The low correlation value at the lowest spatial frequencies could be due to the poor contrast in the edge of the Siemens star. The area with spatial frequencies shaded in blue corresponds to the region of the radial markers in the Siemens star pattern at a radius of 1 mm with features from $130\,{\upmu }\hbox {m}$ to $113\,{\upmu }\hbox {m}$. The resolution of the image was determined with the half-bit threshold criterion^44^ to be $\approx 130\,{\upmu }\hbox {m}$. The experimental results match very well with the simulations shown in Fig. 3b.

## Fresnel zone plates for small angle neutron scattering

Small angle neutron scattering (SANS) is an ideal tool for studying the structure of materials in the mesoscopic size range between 1 and about 400 nm^45–47^. It is a non-destructive method providing structural information averaged over all grains of different sizes with high statistical accuracy due to averaging over the whole sample volume. Using neutrons as probing particles has the advantage of being sensitive to magnetic spins, allowing to resolve magnetic structures, and also to light elements, in particular hydrogen/deuterium which remain invisible in X-ray small angle scattering. The last property is crucial for many applications in biology and polymer research.

**Fig. 7:**
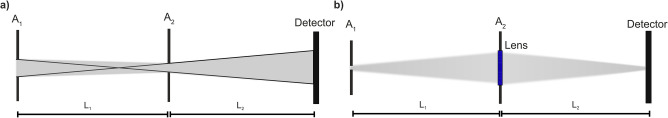
(**a**) Scheme of pinhole collimation geometry SANS instrument. (**b**) Scheme of a Focusing SANS instrument with a lens producing an 1:1 image of the $A_1$ on the detector.

In a typical SANS beamline, a well-collimated neutron beam with a bandwidth of typically $\Delta \lambda /\lambda = 0.1$ incident on the sample is scattered and detected by a position-sensitive detector several meters downstream of the sample. The beam collimation is determined by the initial aperture $A_1$ at $z = 0$, sample aperture $A_2$ at $z=L_1$, see Fig. 7a. An optimal matched collimation condition of $A_2=A_1/2$ creates a truncated cone illumination on the detector with a base diameter of $S= 2 A_1$. This intense direct beam comprising the umbra and penumbra of $A_1$ needs to be eliminated with a beam stop, thereby limiting the smallest momentum transfer $Q_{min}$ that can be observed^10,48,49^:

9 $$\begin{aligned} Q_{min}[Pinhole]= \frac{2\pi }{\lambda }\frac{S}{2L_2} = \frac{2\pi }{\lambda }\frac{A_1}{L_2} \end{aligned}$$

This situation can be improved by the use of a lens, in a modified focusing SANS geometry, with a small $A_1$, and a lens placed upstream of the sample which focuses the neutrons on the detector. An ideal lens in this geometry would produce an image of $A_1$, thereby allowing access to smaller *Q* values while also increasing the neutron flux on the detector, see Fig. 7b.

10 $$\begin{aligned} Q_{{min}}[Lens] = \frac{\pi }{\lambda } \frac{A_1}{L_2} \end{aligned}$$

To improve the resolution and flux several types of focusing elements have been developed, including compound refractive lens (CRL)^8,10,11,50^, mirrors^5^, magnetic lenses^51^ and Wolter optics^7,52,53^. To date, CRLs are commonly used in SANS beamlines. The focal length of a parabolic CRL is given by,

11 $$\begin{aligned} F_{CRL} = \frac{R}{2 N \delta } \propto \lambda ^{-2}\end{aligned}$$

where *N* is the number of lenses and *R* is the apex radius of the parabola. Due to limitations in machining techniques preventing very small *R*, multiple lenses are stacked to achieve the equivalent effect. The thickness profile of a parabolic lens along the radius *r* is given by $T(r) =r^2/(2 R)$. Thus, to double the aperture of the lens, one needs to quadruple the outer thickness of the lens, resulting in poor transmission of the CRLs. This limits the aperture of the CRLs that effectively can be used. Also, the $\lambda ^{-2}$ scaling of the focal length of the CRLs along with the gravity contributions introduces additional blurring to the image of $A_1$ produced by the lens as shown in Fig. 10a. To achieve the maximum possible intensity gain, cooling the CRLs has been suggested to reduce the incoherent scattering from the CRLs^50^.

These challenges could be addressed with a FZP as the focusing element. The $f_{FZP} \propto \lambda ^{-1}$ allows the FZP to have reduced chromatic blurring compared to a CRL where $f_{CRL} \propto \lambda ^{-2}$. Small thicknesses of the FZPs allow them to have negligible absorption and a low inelastic scattering background compared to a CRL. At longer wavelengths, the larger aperture FZPs can be made within the limitations of lithography techniques without considerable absorption losses. These factors make FZPs an attractive candidate as the focusing optics for SANS.

### Fabrication of FZPs for SANS

For a proof-of-principle experiment using the SANS-I beamline at the SINQ spallation source at PSI^54^, we designed a FZP with a diameter of *D* = 16 mm and a focal length of $f_{FZP}$ = 9 m at 12.5 Å  wavelength. The outermost zone width was $\Delta r$ = 703 nm. Of the choices of materials that can be conveniently nanopatterned, silicon was found to be suitable, as the required thickness of the zone structures of $\Delta T_{\pi }=12.1\,{\upmu }\hbox {m}$ are achievable by DRIE. The parameters of the silicon FZP used are listed in Supplementary Table 1.

**Fig. 8:**
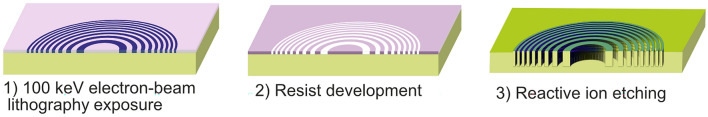
Fabrication steps of a silicon FZP by electron-beam lithography and deep reactive ion etching.

The silicon FZPs were fabricated using electron beam lithography following the steps shown in Fig. 8. A 18 mm $\times$ 18 mm silicon substrate of $250\,{\upmu }\hbox {m}$ thickness with 800 nm thick SiO$_2$ on top was spin coated with an 11% solution of PMMA 950K in anisole at 3800 rpm, and then baked at 175$^{\circ }$C for 5 minutes resulting in a thickness of $2\,{\upmu }\hbox {m}$. The pattern was exposed with a 100 keV electron beam using a Vistec EBPG 5000+ system. The exposed resist was developed in room temperature 7:3 IPA:H$_2$O solution. Then the pattern is transferred in the SiO$_2$ layer and then etched in the silicon wafer using the DRIE process. The process was optimized to yield vertical side walls and uniform etch depth.

**Fig. 9:**
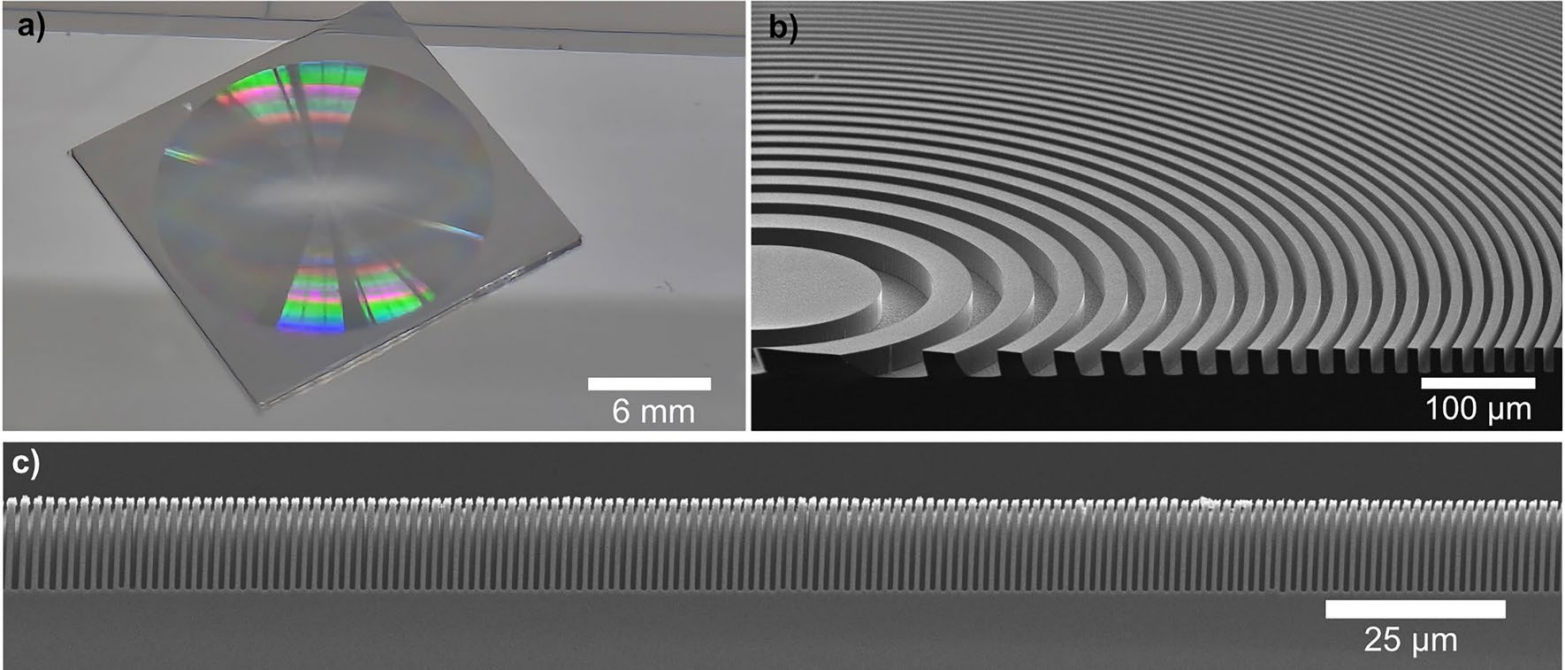
(**a**) Photograph of a 16 mm diameter silicon FZP, (**b**) Tilted cross-section SEM image of central zones of a silicon FZP, (**c**) Cross-section SEM image of outer zones of a silicon FZP.

A resulting FZP is shown in Fig. 9. To inspect the depth and profile of the zone structures, a FZP was cleaved and imaged in the SEM. The quality of the silicon FZP is higher compared to the nickel FZP used for imaging, which is due to the more relaxed outermost zone width. In particular, as can be seen from Fig. 9c, the depth of the zones matches the ideal value of $T_{\pi } =12.1\,{\upmu }\hbox {m}$ and the duty cycle is close to the desired value of $50 \%$ . It can therefore be expected that the silicon FZP has an efficiency of $\approx 40 \%$ at 12.5 Å  wavelength, as shown in Supplementary Fig. 1b.

### SANS experiments

The silicon FZP was mounted at the 40 m long SANS-I instrument. It is supplied with neutrons through a neutron guide from the SINQ source. A velocity selector downstream the neutron guide with 10$\%$ bandwidth was set to 12.5 Å  central wavelength. The initial aperture $A_1$ with a diameter of 2 m is situated downstream the velocity selector. The 16 mm diameter silicon FZP was mounted on a 16 mm diameter Cd aperture $A_2$ and placed on the sample stage at 18 m downstream of the $A_1$. A two-dimensional position sensitive $^{3}$He-detector (PSD) with 128 $\times$ 128 pixel with 7.5 mm $\times$ 7.5 mm pixel size was placed in a vacuum vessel at 18 m downstream of the FZP. In addition, a neutron imaging plate was mounted for high-resolution neutron detection. The exposed image plate was later read out with a GE Typhoon FLA 7000 Fluorescence Imaging System into a 16-bit file.

**Fig. 10:**
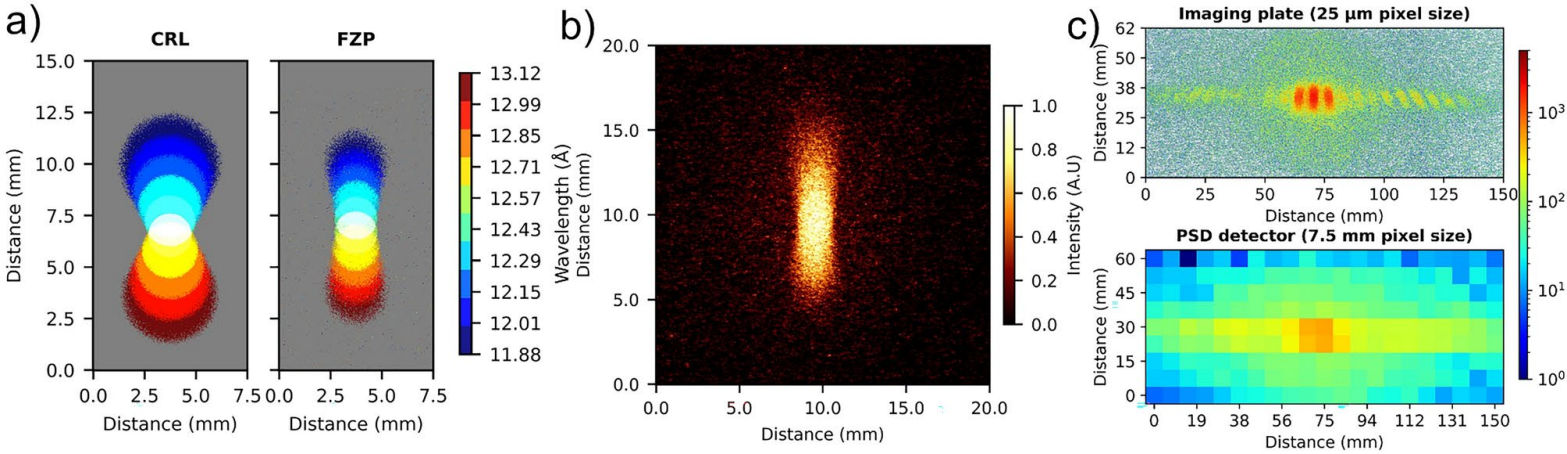
(**a**) McStas simulation of imaging an aperture with $A_1$ = 2 mm diameter using a CRL and a FZP at 12.5 Å   wavelength with 10% bandwidth and distances of $L_1$ = $L_2$ = 18 m. The contribution due to different wavelengths are color-coded. (**b**) Image of a 2 mm diameter $A_1$ pinhole formed by the FZP at 12.5 Å  neutron beam at 10 $\%$ bandwidth on an imaging plate. The exposure time was 2400 s. (**c**) Scattering pattern produced by a $3.5\,{\upmu }\hbox {m}$ period silicon grating with the FZP in a focusing SANS geometry in an imaging plate (top) and the PSD detector (bottom). The exposure times were 2100 s and 300 s respectively.

To characterize the performance of the silicon FZP, the $A_1$ = 2 mm aperture was imaged with the silicon FZP as a focusing optic at $L_1=L_2=$18 m. The 1:1 image of $A_1$ is smaller than the $7.5 \times$ 7.5 mm pixel size of the PSD. Therefore, the high-resolution neutron imaging plate was used to characterize the performance of the silicon FZP. The gravity effects combined with the chromaticity of the FZP distorted the 2 mm pinhole image to a bow-tie structure as shown in the simulations, see Fig. 10a. The center of the bow-tie corresponds to the 12.5 Å  neutrons in focus dropped down by $\approx$ 16 mm from the beam axis. The vertical elongation of the circle $\approx$ 3 mm above and below correspond to the shorter and longer wavelength neutrons in the beam respectively, see Fig. 10b. The horizontal elongation corresponds to the defocused part in the image of $A_1$ due to the shorter and longer neutron wavelengths than the design wavelength of the FZP.

To demonstrate that the silicon FZP is capable of resolving low-Q values, a strongly scattering silicon grating of pitch $3.5\,{\upmu }\hbox {m}$ was placed at a short distance upstream of the FZP. The scattering pattern was recorded at $L_2$ = 18 m with the PSD detector and an imaging plate as shown in Fig. 10c. The diffraction peaks—most notably the intense $-1st$, zero, and $+1st$ diffraction order of the grating—can be resolved when using the high-resolution imaging plate detector. The grating pitch corresponds to a momentum transfer $1.8\times 10^{-4}$ Å$^{-1}$. The contribution of gravity again elongates the diffraction peaks and the dispersion from the grating makes them slanted. It should be noted that the chromatic aberration of the FZP does not affect the scattering pattern.

When recording the grating scattering pattern with the PSD, the diffraction peaks cannot be resolved due to the large pixel size. However, a significant level of diffuse scattering can be observed in the vicinity of the direct beam (meaning the image of $A_1$). The size of this diffuse scattering region corresponds well to the expected size of the diverging $-1st$ diffraction order of the FZP of 64 mm. As the efficiency of the $-1st$ and $+1st$ diffraction order should be identical, we estimate that the background contains $\approx 40 \%$ of the incoming intensity.

## Discussion

In the previous section, we demonstrated the use of FZPs for proof-of-concept experiments in full-field neutron microscopy and SANS. The advanced nanofabrication techniques originally developed for X-ray diffractive optics have been successfully adapted for neutron-specific requirements. While X-ray FZPs are typically fabricated with diameters of a few $100\,{\upmu }\hbox {m}$, we optimized the electron beam lithography and the pattern transfer techniques to produce neutron FZPs with significantly larger diameters, as shown in Figs. 5 and 9. These larger diameters are essential for efficiently utilizing the low flux at neutron sources.

The nickel FZP used for full-field neutron microscopy featured a diameter of 1.2 mm, an outermost zone width of 372 nm, and a thickness of approximately $3\,{\upmu }\hbox {m}$, resulting in an aspect ratio of about 8. For our neutron imaging experiments, we demonstrate that it is possible to mechanically stack two of these elements to achieve a diffraction efficiency close to the theoretical maximum of $40.5\%$. In the future, we aim to overcome the limitations of mechanical stacking by using a multi-step electron beam lithography approach^6,55,56^, by achieving a precise (<25 nm) on-chip alignment for enabling high-aspect-ratio FZPs ($AR>15$). This advancement will simplify the alignment at the neutron instrument and potentially allow for neutron FZPs with an outermost zone width as small as 100 nm, thereby enabling shorter focal lengths and higher imaging magnifications of the sample. Despite these advancements, the spatial resolution achieved in our full-field neutron microscopy experiments was far from the diffraction-limited value described in Equation 6 due to the chromatic aberration of FZPs. To reduce the chromatic blurring, the neutron beam needs to be monochromatized using either a crystal monochromator or a velocity selector at the cost of severely reduced neutron flux. Alternatively, an achromatic neutron lens could be realized by combining a neutron FZP with a neutron compound refractive lens^57^, two types of optics that have different dispersive properties. Such achromatic lenses have been demonstrated for X-rays^58^ and could represent a significant advancement in full-field neutron microscopy using broadband radiation. However, realizing such achromatic optics for neutrons presents significant challenges, primarily due to the selection of materials, fabrication of the two types of lenses, and their relative alignment. Such achromatic optics would enable efficient focusing and imaging with broadband sources, minimizing chromatic aberration. Lens-based instruments with such efficient lenses could achieve better spatial resolution compared to pinhole-based instruments that are constrained by a trade-off between the exposure time and the resolution, ultimately limited by the pixel size of the camera and the thickness of the scintillator.

The FZPs for small-angle neutron scattering were fabricated from silicon. They had a diameter of 16 mm, an outermost zone width of 703 nm, and a thickness of $12\,{\upmu }\hbox {m}$, which provided optimal diffraction efficiency for the neutron wavelength of 12.5 Å. This fabrication method was chosen because deep reactive ion etching achieves the necessary aspect ratio of 17 for silicon at a 703 nm width, eliminating the need to produce and stack two individual elements, as was required for nickel FZPs.

The silicon FZPs were successfully used to achieve focused neutron beams for small-angle neutron scattering and to enable the measurements of low-Q values down to $10^{-4}$ Å$^{-1}$ range. However, an ideal lens for a SANS instrument must not only focus effectively but also have a low parasitic scattering contribution to provide a signal-to-background ratio better than $10^4$. In our proof-of-concept experiment, the silicon FZP achieved a high efficiency of approximately $40.5\%$, but it also produced a divergent $-1^{st}$ order diffraction resulting in excessive diffuse background. In the future, modifying the binary FZP to a blazed profile could improve efficiency up to $81\%$ at the focal point^59,60^ while strongly reducing the background signal because the divergent $-1^{st}$ order would have a much reduced diffraction efficiency. Thus, this modification would allow the FZP to enable the measurement of lower-Q scattering with a higher signal-to-noise ratio. Last, due to gravitational effects at 12.5 Å  wavelength, neutrons fell approximately 16 mm, which caused gravitational blurring and impacted instrument resolution, particularly in the vertical direction. In the future, this effect could be minimized by either using shorter wavelengths or reducing the focal length of the FZP, $f_{FZP}$, to shorten the distance to the detector, $L_2$. Finally, neutron FZPs for SANS offer two distinct advantages over compound refractive lenses: (1) FZPs can be fabricated with larger diameters without suffering from excessive absorption as it occurs for CRL with large apertures and (2) FZPs exhibit a reduced chromatic aberration, as demonstrated in Fig. 10a.

## Conclusions

Diffractive optical elements are well-established focusing and imaging devices that are particularly suitable for X-rays due to their versatility, compact form factor and easiness of use. In the recent years, advanced high-resolution lithography and nanofabrication techniques have been exploited for producing high-quality diffractive optics for X-ray beams. In this work, we showed that these nanofabrication methods can be readily extended to realize diffractive lenses for neutron-based applications. We demonstrate that Fresnel zone plates made of nickel and silicon can be used for full-field neutron microscopy and SANS experiments. These demonstrations highlight the promising potential of neutron diffractive optics. In the future, further advancing the nanofabrication methods for neutron diffractive optics with high aspect ratio or blazed profiles will pave the way for the advancing applications in neutron imaging, focusing and beam delivery.

## Supplementary Information

Supplementary Information.

## Data Availability

The datasets used and/or analysed during the current study available from the corresponding author on reasonable request.
