# Supplementary material for: Diffractive lenses for neutron techniques
Source: Sci Rep. 2025 Mar 11;15:8408. doi: 10.1038/s41598-025-92329-6 (PMC11897255; doi:10.1038/s41598-025-92329-6)
Supplement: Supplementary file 1 — Supplementary Information. [file 41598_2025_92329_MOESM1_ESM.pdf]

Supplementary Information for "Diffractive lenses for neutron techniques"

Mano Raj Dhanalakshmi Veeraraj[1,*], Di Qu[1], Shuai Zhao[1,4], Peng Qi[1],
Konstantins Jefimovs[1], Matteo Busi[2], Joachim Kohlbrecher[2],
Christian David[1], Markus Strobl[2,3], Joan Vila-Comamala[1]

[1]PSI Center for Photon Science, Paul Scherrer Institute, Villigen PSI, 5232, Switzerland
[2]PSI Center for Neutron and Muon Sciences, Paul Scherrer Institute, Villigen PSI, 5232, Switzerland
[3]Niels Bohr Institute, University of Copenhagen, København, 2100, Denmark
[4]National Synchrotron Radiation Laboratory, University of Science and Technology of China, 230029 Hefei, China

[*]Corresponding author: mano.dhanalakshmi-veeraraj@psi.ch

**Experiment**	**Full field microscopy**	**Small angle scattering**
**Material**	Nickel	Silicon
**Diameter (mm)**	1.2	16
**λ (Å)**	4.5	12.5
**ΔT_π (μm)**	7.4	12.1
**ΔT_{exp} (μm)**	6	12
**δ**	3.03×10^{-5}	5.14×10^{-5}
**Theoretical efficiency (%)**	36.7	40.5
**Number of zones**	805	5689
**Δr (nm)**	372	703
**F_{FZP} (m)**	0.99	9

Supplementary Table 1: Parameters for the Ni and Si FZP designed for the BOA and SANS 1 beamlines.

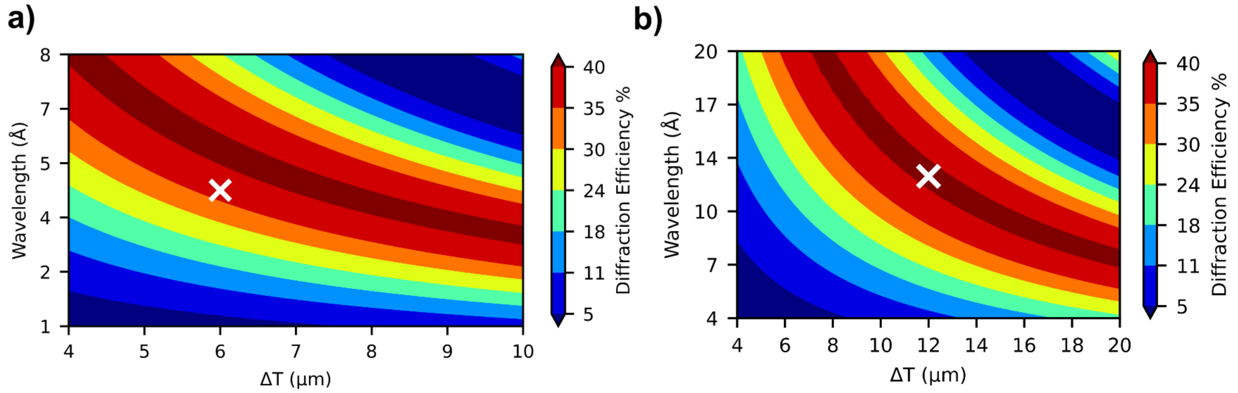

Supplementary Figure 1: (a) Efficiency map of Ni FZP used as an objective lens in the Full field neutron microscopy experiment. The white cross corresponds to the ΔT_{exp} =6 µm of the FZPs used in the experiment. (b) Efficiency map of the Si FZP used as a focusing lens in SANS experiment. The white cross corresponds to the $\Delta T_{\mathrm{exp}} = 12$ µm of the FZP used in the SANS experiment.

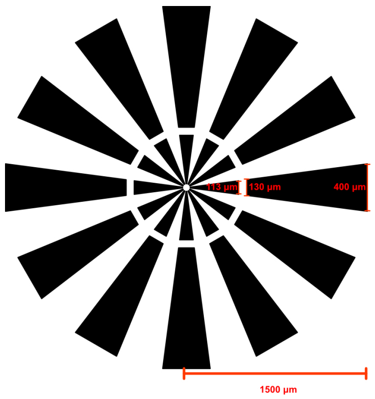

Supplementary Figure 2: 3 mm diameter Siemens star with annotations of some dimensions.

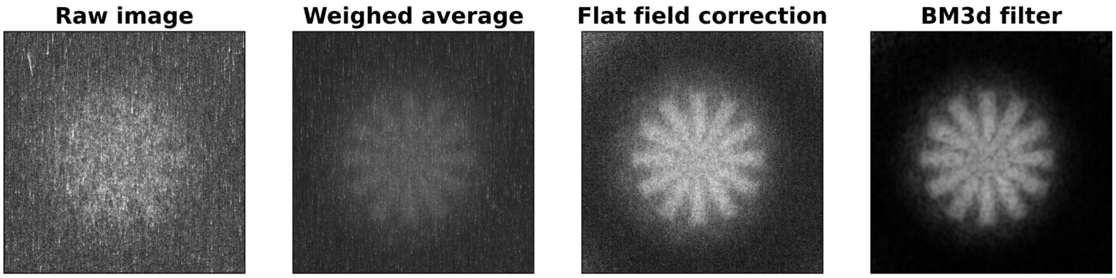

Supplementary Figure 3: Steps in image processing, involving spot removal by weighted average, flat field correction, and noise reduction using BM3D filter.
